# Apoptosis-related gene expression can predict the response of ovarian cancer cell lines to treatment with recombinant human TRAIL alone or combined with cisplatin

**DOI:** 10.6061/clinics/2020/e1492

**Published:** 2020-03-09

**Authors:** Letícia da Conceição Braga, Nikole Gontijo Gonçales, Rafaela de Souza Furtado, Warne Pedro de Andrade, Luciana Maria Silva, Agnaldo Lopes da Silva Filho

**Affiliations:** I Servico de Biologia Celular da Diretoria de Pesquisa e Desenvolvimento, Fundacao Ezequiel Dias-Funed, Belo Horizonte, MG, BR; II Departamento de Ginecologia e Obstetricia, Faculdade de Medicina, Universidade Federal de Minas Gerais, Belo Horizonte, MG, BR; III Departamento de Ginecologia e Obstetricia, Faculdade de Ciencias Medicas e Biologicas de Botucatu, Universidade Estadual Paulista, Botucatu, SP, BR

**Keywords:** Ovarian Cancer, Gene Expression, Chemoresistance, Apoptosis, Trail

## Abstract

**OBJECTIVES:**

The objectives of this study were to determine the sensitivity of ovarian cancer (OC) cell lines (TOV-21G and SKOV-3) to cisplatin and to the recombinant human TRAIL (rhTRAIL), and to evaluate the expression profile of *TNFRSF10B, TNFRSF10C*, *TP53TG5, MDM2, BAX, BCL-2 and CASPASE-8* genes and their participation in the resistance/susceptibility mechanism of these tumor cell lines.

**METHODS:**

To determine the IC_50_ values associated with Cisplatin and rhTRAIL, inhibition of cell growth was observed using MTT assays in two human OC cell lines (SKOV-3 and TOV-21G). The analysis of gene expression was performed using quantitative real-time polymerase chain reaction (qRT-PCR).

**RESULTS:**

Both cell lines had different susceptibility profiles to the tested drugs. In the SKOV-3 cell line, the IC_50_ values for cisplatin and for rhTRAIL were 270.83 ug/mL and 196.5 ng/mL, respectively. The same concentrations were used for TOV-21G. Different gene expression profiles were observed in each tested cell line. *CASPASE*-8 and *TNFRSF10B* expression levels could predict the response of both the cell lines to rhTRAIL alone or the response to a combination of rhTRAIL and cisplatin. In addition, we observed a relationship between *BCL-2* and *BAX* expression that may be helpful in estimating the proliferation rate of the OC cell lines.

**CONCLUSION:**

SKOV-3 and TOV-21G respond differently to cisplatin and rhTRAIL exposure, and expression of *CASPASE*-8 and *TNFRSF10B* are good predictors of responses to these treatments.

## INTRODUCTION

Ovarian cancer (OC) is a highly fatal form of gynecological cancer characterized as the 7^th^ most common cause of cancer in women worldwide (1,2). In 2018, OC was estimated to be responsible for 295,414 new cases of cancer and 184,799 deaths worldwide (3). In Brazil, OC is 8^th^ most common type of cancer in women. The estimated number of new OC cases in 2018-2019 was 6,150 (4) with an associated risk of 5.79 cases per 100,000 women.

The high mortality rates of OC is partially due to the inefficient detection of the disease in its initial stage, resulting in the diagnosis of 75% of the patients in the advanced stages (5). Chemotherapy involving platinum and taxanes has been used to treat OC for the last four decades. Although most patients show an initial response to treatment, the disease recurs within two years of treatment in 60%-80% of the patients (6). The major factor that limits the efficiency of chemotherapy is resistance acquisition (7). Some mechanisms proposed to contribute to resistance include decreased drug capture by cells, increased drug efflux, and increased DNA repair (8). In addition, characteristics such as self-renewal, chemoresistance, anchorage-independent growth, and apoptosis resistance have been associated with drug resistance, cancer progression, and tumor recurrence (9-11). However, the molecular mechanisms underlying resistance to chemotherapy have not been fully understood (12). Recent advances in adjuvant chemotherapy for OC, such as combining platinum with paclitaxel, have increased patient survival, however complete remission remains rare, and the success of recurrent cancer treatments is limited. Thus, new therapeutic modalities for the treatment of OC are necessary (13,14).

The TRAIL (TNF-related apoptosis-inducing ligand) receptor pathway has been a focus of studies that are aimed at developing a new strategy to combat OC resistance to cisplatin treatment (15). TRAIL can selectively induce apoptosis in tumor cells with little toxicity to normal cells (16). The TRAIL protein can attach to four cell-surface receptors. TNFRSF10A (TRAIL-R1/DR4) and TNFRSF10B (TNFRSF10B/DR5) induce apoptosis, however TNFRSF10C (TNFRSF10C/DcR1) does not contain an intracytoplasmic death domain and TNFRSF10D (TRAIL-R4/DcR2) contains a truncated death domain, resulting in the latter two being unable to transmit apoptotic signals (17). Data on the involvement of these receptors in apoptosis resistance in OC remain scarce. Despite the heterogeneity of molecular profiles and histological phenotypes of OC, most cases are treated indiscriminately (16,18). The present study aims to determine the sensitivity of OC cell lines (TOV-21G and SKOV-3) to cisplatin and to the recombinant human TRAIL (rhTRAIL), to evaluate the expression of TRAIL receptors (*TNFRSF10B* and *TNFRSF10C*), repair genes (*TP53TG5* and *MDM2*), and apoptotic cascade genes (*BAX*, *BCL*-2, and *CASPASE*-8) in these cell lines, and to analyze their participation in drug resistance and drug susceptibility.

## METHODS

### Cell Lines

TOV-21G (Cat. #CRL-11730™) and SKOV-3 (Cat. #CRL-7566™) were acquired from the American Type Culture Collection (ATCC, Manassas, VA, USA). TOV-21G was cultivated in the Dulbecco's Modified Eagle's Medium - High Glucose, supplemented with 15% fetal bovine serum. The SKOV-3 line was cultivated in the McCoy's 5A (Modified) medium supplemented with 10% fetal bovine serum. Both cell lines were incubated at 37°C with 5% CO_2._

### Cell Survival Analysis

The TOV-21G and the SKOV-3 cell viabilities in the presence of cisplatin and rhTRAIL were determined using the 3-(4,5-Dimethylthiazol-2-yl)-2,5-Diphenyltetrazolium Bromide (MTT) method. Cells were plated in 96-Well cell culture plates (1.0x10^5^ cells/well) in triplicate and incubated for 24h at 37°C with 5% CO_2_. After 24h, the wells were washed with 1X PBS and the cells were treated with varying concentrations of cisplatin (275 ug/mL-100 ug/mL) and rhTRAIL (600 ng/mL-0.78 ng/mL). Non-treated cells were used as experimental controls. After 24h, MTT solution was added to the plate (0.5 mg/mL final concentration), cells were incubated for 3h and the absorbance at 540 nm was measured using a Spectramax M5e. The concentration of each drug that inhibited the viability of 50% of the cells (IC_50_) from each cell line was calculated using an Origin 8.5.1 software.

### Total RNA extraction and reverse transcription

Total RNA was extracted from untreated control cells and cells treated with cisplatin, rhTRAIL or a combination of each drug (IC_50_) using TRIzol^®^ (Invitrogen TM, USA) according to the manufacturer’s instructions. The total RNA concentration and the 260/280 absorbance ratio were measured using the NanovueTM Plus Spectrophotometer (GE Heathcare Life, USA) microvolume spectrophotometer, and RNA integrity was evaluated by 1% agarose gel electrophoresis. Total RNA was then treated with RNase-free DNase Set^®^ (Qiagen) and 2 μg of RNA was used for complementary DNA (cDNA) synthesis using the M-MLV Reverse Transcriptase^®^ kit (Sigma), according to the manufacturer’s instructions.

### Gene expression analysis

The expression of *TNFRSF10B, TNFRSF10C, TP53TG5, MDM2, BAX, BCL-2,* and *CASPASE-8* genes was evaluated by quantitative real time polymerase chain reaction (qRT-PCR), using the PowerUp™ SYBR^®^ Green Master Mix, according to the manufacturer’s instructions. *TBP* and *RPS26* genes were included as control genes for normalization. The primers used in this study have been shown in Table 1.

Control samples representing total RNA without reverse transcription were included in each assay. PCR reactions followed the conditions outlined in Table 1 according to the manufacturer’s instructions. Data was collected using the Mx3005P^®^ qPCR System (Stratagene). The 2(-Delta C(T)) method (16) was used to quantify the relative expression levels of each target gene.

### Statistical Analysis

Data were analyzed using the SPSS 18.0 software package (SPSS Inc., Chicago, Illinois, USA). Differences in the gene expression were statistically evaluated using the non-parametric Kruskal-Wallis and the Levene tests. Differences were considered statistically significant when *p*<0.05. Clustering analysis was performed using the UPGMA method and the Euclidian distance was calculated using the BioNumerics software version 7.5 (Applied Maths, Sint-Martens-Latem, Belgium).

## RESULTS

### Cell Viability Analysis

**Figure 1:**
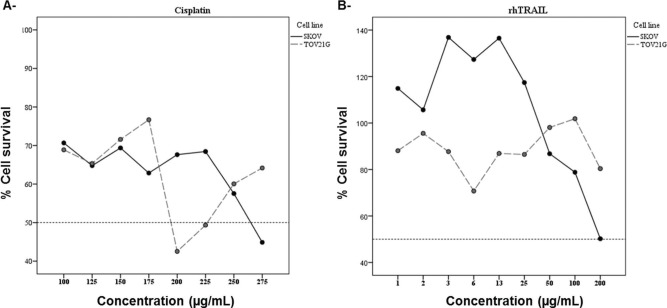
Viability of the SKOV-3 and the TOV-21G cell lines in response to different concentrations of cisplatin (1A) and rhTRAIL (1B) tested. The concentrations of cisplatin used varied from 100 ug/mL to 275 mg/mL. The concentrations of rhTRAIL used varied from 0.78 mg/ml to 200 ng/ml.

MTT assays were used to evaluate the effects of cisplatin and rhTRAIL (24-h exposure) on the viability of the SKOV-3 and the TOV-21G cell lines. All cisplatin concentrations tested reduced the viability of SKOV-3 cells, with the IC_50_ being 275 ug/mL (Figure 1A). Concentrations of rhTRAIL in the range of 0.78 ng/mL to 25 ng/mL permitted cell proliferation, while concentrations above 50 ng/mL inhibited cell growth, with an IC_50_ close to 200 ng/mL (Figure 1B). Using the Origin statistics program, IC_50_ values were calculated as 270.83 ug/mL for cisplatin and 196.5 ng/mL for rhTRAIL. In addition, all tested concentrations of cisplatin reduced the TOV-21G cell viability, despite variation in the assays (Figure 1A). Although most of the tested rhTRAIL concentrations reduced the TOV-21G cell viability, none inhibited cell growth by 50% (Figure 1B). Thus, we decided to use the IC_50_ values associated with cisplatin and rhTRAIL treatment of the SKOV-3 cells for experiments with TOV-21G cells in order to compare the effects of these drugs on each cell line.

Different ratios of cisplatin and rhTRAIL at IC_50_ were used to evaluate the effect of combining these drugs on the SKOV-3 cell viability. Upon treatment with a 1:1 ratio of cisplatin:rhTRAIL, the cell viability was found to be 28.49%. This corresponded to a strong synergistic effect, since the cell density was almost half of that observed when the drugs were used separately. The IC_50_ associated with combining the drugs could not be statistically calculated. Thus, in order to evaluate the effect of combined cisplatin-rhTRAIL treatment on gene expression, we opted to consider the IC_50_ of the SKOV-3 cells to correspond to a 2:2 cisplatin:rhTRAIL ratio, since this produced ∼50% cell viability (44.58%) (Table 2).

### Gene Expression Analysis

**Figure 2:**
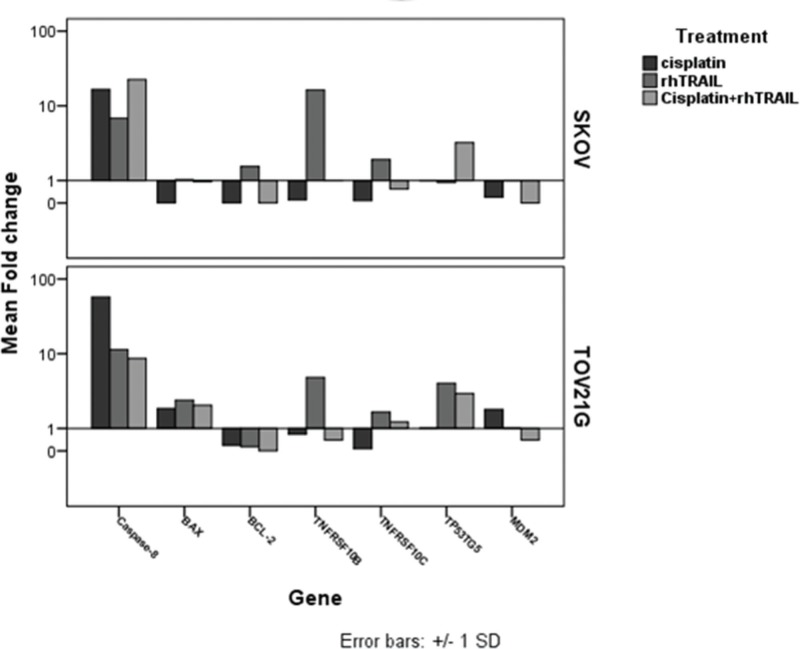
Relative expression analyses of the *TNFRSF10B, TNFRSF10C, TP53TG5, MDM2, BAX, BCL-2 and CASPASE-8* genes in the TOV-21G AND the SKOV-3 cells treated with cisplatin and rhTRAIL alone, or in combination. Gene expression profiles were different in both cell lines treated with rhTRAIL alone. Treatment with cisplatin alone or in combination with rhTRAIL led to differences in the expression of *CASPASE-8* or *BAX* genes, respectively.

We analyzed the expression of seven genes (*TNFRSF10B, TNFRSF10C, TP53TG5, MDM2, BAX, BCL-2* and *CASPASE-8*) following treatment of each cell line with cisplatin or rhTRAIL alone, or treatment with a combination of cisplatin and rhTRAIL. The SKOV-3 cells exhibited down-regulation of all genes studied in response to each treatment, with a few exceptions: the rhTRAIL treatment resulted in the up-regulation of *TNFRSF10B* and *CASPASE*-8, while cisplatin and combined treatments resulted in the up-regulation of *TP53TG5* (Figure 2A).

The TOV-21G cells were characterized by a different gene expression profile than that used for the SKOV-3 cells. In TOV-21G cells treated with cisplatin alone, only *TP53TG5* and *CASPASE-8* were up-regulated, while in presence of rhTRAIL alone or combined with cisplatin, *CASPASE*-8 and *BAX* were up-regulated and *BCL-2* was down-regulated (Figure 2B). In addition, *TP53TG5* was up-regulated while *M2D2* was down-regulated in the presence of cisplatin alone and in combination with rhTRAIL in both cell lines (Figures 2A and 2B).

Thus, different gene expression profiles were observed between both cell lines treated with rhTRAIL alone. Similar profiles were observed for all genes when the cells were treated with cisplatin alone or combined with rhTRAIL, except for *CASPASE-8* and *BAX*, respectively.

**Figure 3:**
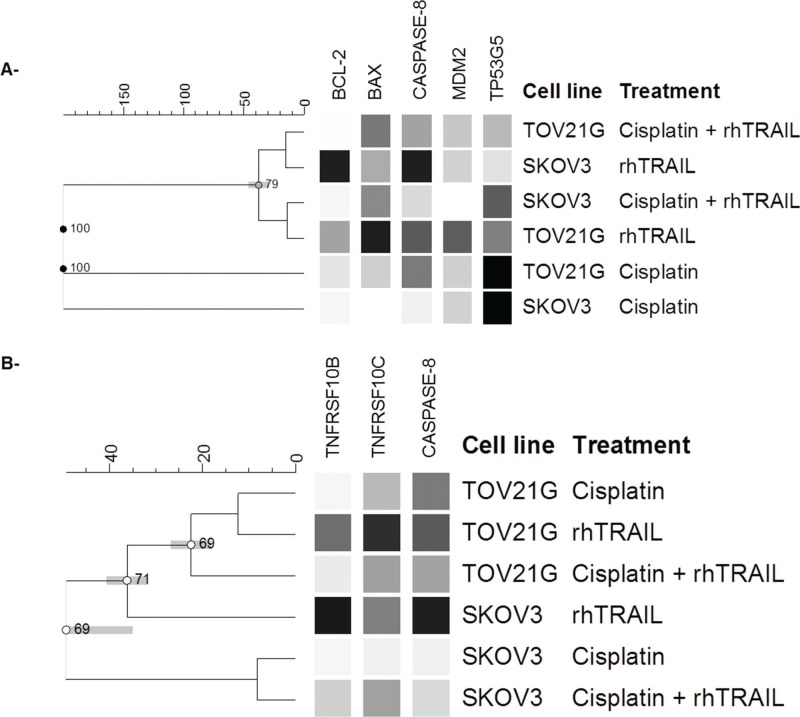
Cluster analysis of *TNFRSF10B, TNFRSF10C, TP53TG5, MDM2, BAX, BCL-2 and CASPASE-8* genes in the TOV-21G and the SKOV-3 cells treated with cisplatin or rhTRAIL alone, or in combination. Following cisplatin treatment, *TP53TG5* was up-regulated in both cell lines, while *CASPASE-8* was up-regulated in the TOV-21G cells. Following rhTRAIL treatment, *CASPASE-8* and *TNFRSF10B* up-regulation was observed in the SKOV-2 cells and *CASPASE*-8 up-regulation was observed in the TOV-21G cells. Combined cisplatin-rhTRAIL treatment up-regulated *CASPASE*-*8* in the TOV-21G cells.

Clustering of the gene expression results observed for the TOV-21G and the SKOV-3 cell lines highlighted genes that revealed molecular signatures specific to each cell line and treatment type. Upon cisplatin treatment, we observed increased expression of *TP53TG5* in both cell lines (Figure 3A). However, the expression of *TP53TG5* was approximately 100-fold higher in TOV-21G cells compared to that in the SKOV-3 cells. Increased expression of *TNFRSF10B* and *CASPASE-8* in the SKOV-3 cells and *CASPASE-8* in the TOV-21G cells were highlighted as molecular markers of the rhTRAIL treatment response (Figure 3B). When considering the combination of cisplatin and rhTRAIL, *CASPASE-8* may be highlighted as a molecular marker of the synergistic effect observed on the TOV-21G cell viability (Figure 3B).

## DISCUSSION

OC comprises a set of neoplasms with distinct clinicopathological and molecular features and clinical outcomes. Recently, studies on molecular genetics have led to the development of a dualistic model (Type I *vs*. Type II) for the characterization of Epithelial OC (EOC) (20). Type I typically represents large tumors confined to the ovary that are indolent and are associated with good prognosis. They are relatively genetically stable and rarely contain *TP53* mutations. Type II tumors are normally advanced (stages III-IV) when diagnosed. They are characterized by rapid growth and are extremely aggressive, with high chromosomal instability and mutations in *TP53* in >95% of the cases (20). *TP53* is a tumor suppressor gene that encodes a transcription factor involved in cellular stress responses which is activated in response to DNA damage by genotoxic agents.

Despite the heterogeneity observed in OC, all cases are treated as a single disease (20,21). The standard treatment is an invasive surgery followed by platinum-taxane chemotherapy. Platinum-resistance occurs in many patients, therefore the chance of survival past five years is ∼30% (22). Recently, efforts have been made to identify biomarkers for new therapeutic strategies to overcome current therapeutic limitations.

In this study, we used the SKOV-3 and the TOV-21G cell lines as models of EOC in order to study the effects of cisplatin and rhTRAIL on the apoptosis-related gene expression. TOV-21G is derived from a primary clear cell carcinoma (grade 3, stage III), contains the wild-type *TP53*, and is representative of a type I tumor. SKOV-3 originates from metastatic cells (ascites) of an ovary adenocarcinoma and is characterized by platinum-resistance, mutations in *TP53,* thus representing a type II tumor.

Differences in cell viability were observed in both cell lines treated with cisplatin and rhTRAIL. The SKOV-3-associated IC_50_ values were calculated as 270.83 ug/mL for cisplatin and 196.5ng/mL for rhTRAIL. However, variation in cell viability was observed upon treatment of the TOV-21G cells with cisplatin or rhTRAIL, therefore the IC_50_ values could not be accurately determined. It was not possible to observe the dose-response effects, as increased drug concentration did not affect the cell viability proportionally. Karbownik et al. (23) tested the physical and chemical stability of cisplatin (1 mg/mL), using High-Performance Liquid Chromatography (HPLC) for 30 days, and observed that the average concentration decreased to 92.09%. Considering that more than 30 days of drug storage was necessary for the establishment of the TOV-21G cell cultures and performance of cytotoxic assays, changes in the cisplatin stability may explain the results obtained by the MTT assay.

Gene expression analyses in both cell lines revealed a heterogeneous transcriptional activity of *TNFRSF10B, TNFRSF10C, TP53TG5, MDM2, BAX, BCL-2* and *CASPASE-8*, which may reveal functional features of the SKOV-3 and the TOV-21G cell lines that may explain our results. High expression of *TP53TG5* was observed in the TOV-21G cells, corresponding to a 100-fold increase compared to the levels observed in the SKOV-3 cells. *TP53* regulates the expression of *TP53TG5*, a negative regulator of cell growth that functions in cell cycle arrest (35,36). Activated *TP53* promotes transactivation of its targets that are implicated in the induction of cell cycle arrest and/or apoptosis, indicating that TP53 plays a critical role in the DNA damage response (24). The TOV-21G cells express a wild-type *TP53*, and the changes in *TP53TG5* expression observed in these cells after exposure to a genotoxic drug imply that *TP53* is actively involved in repair. This also may explain the variation in cell viability observed in the TOV-21G cells treated with cisplatin.

Upon treatment with rhTRAIL, defective drug capture by the TNFRSF10C and TNFRSF10D TRAIL receptors may explain the failure in determining the IC_50_ for this drug. Our gene expression assays detected *TNFRSF10C* up-regulation in the TOV-21G cells, which corroborates this hypothesis. In addition, *TNFRSF10D* can activate the NF-kB signaling through adapter proteins such as TRAF2 and RIPK1, which interact with the *TNFRSF10D* truncated intracytoplasmic domain. Activation of this pathway has implications in cell survival (16,25). *TNFRSF10D* was not evaluated in this study, however, this concept may have led to the results obtained herein.

The IC_50_ values observed in the SKOV-3 cells may be related to the fact that the mutated TP53 in this cell line was unable to perform repair. Thus, the high proliferative rate of these cells may cause the exposure of more cells to chemotherapy. This phenomenon has been described in clinical practice, wherein type II tumors that were more aggressive than type I tumors displayed better responses to chemotherapy due to their rapid growth (20).

Analysis of the gene expression following cisplatin treatment demonstrated up-regulation of *TP53TG5* in the TOV-21G cells (characterized by a wild-type *TP53*). These data suggest *TP53* may have responded to DNA stress by transactivating its targets. It has been reported that TP53 has been involved in the induction of many genes related to apoptosis, including TRAIL death and defectives receptors (26). Almodóvar et al. (27) analyzed the expression of TRAIL receptors in the MCF-7 (*TP53* wild-type) and the EVSA-T (*TP53*-mutated) breast cancer cell lines before and after treatment with doxorubicin. Doxorubicin binds DNA and interrupts replication, a mechanism of action similar to that by cisplatin. In the MCF-7 cells, TNFRSF10A, TNFRSF10B and TNFRSF10C receptor expression was increased upon doxorubicin treatment, whereas this profile was not observed in the EVSA-T cells treated with doxorubicin. This suggests that *TP53* may induce the expression of these receptors in the MCF-7 cells. In our study, increased expressions of TNFRSF10B and TNFRSF10C receptors after cisplatin treatment was not observed. Despite that the TOV-21G cells (*TP53* wild-type) showed lower expressions of TNFRSF10B and TNFRSF10C receptors , other mechanisms may be involved in the control of expression of the TRAIL receptor in ovarian tumors.

Apoptosis is regulated in part by *BCL-2* genes, which promote cell survival and the expression of the pro-apoptotic protein BAX (28). Considering these two targets are antagonistic in the apoptosis pathway, *BAX*:*BCL-2* expression ratios were calculated to define the apoptotic profile of both cell lines in response to cisplatin and rhTRAIL treatments. Ratios greater than one represent a pro-apoptotic profile, while ratios lower than one represent an anti-apoptotic profile (28,29,30). The ratio between *BAX* and *BCL-2* expression in the TOV-21G cells treated with cisplatin was 3.37. This, together with the up-regulation of *TP53* and *CASPASE-8*, demonstrates a pro-apoptosis signaling profile in this cell line, even in the absence of a dose-response effect. In the SKOV-3 cells treated with cisplatin, the *BAX*:*BCL*-2 ratio was 0.64, reflecting an anti-apoptotic profile of this cell line. However, according to ATTC, the SKOV-3 cells were platinum-resistant. The same results were observed with respect to the rhTRAIL treatment and targets related to the TRAIL drug response. The *BAX*:*BCL-2* ratio in the SKOV-3 cells was 0.75; whereas the ratio was 4.86 in the TOV-21G cells, despite that the up-regulation of *TNFRSR10B* in the presence of its ligand was stronger in the SKOV-3 cells than that in the TOV-21G cells. TNFRSF10B is the functional receptor, however it was not expressed sufficiently to induce the SKOV-3 cell death. This may be explained by the fact that mRNA expression levels do not reflect the protein expression on the cell surface (31). In addition, according to ATTC, the SKOV-3 cells were resistant to tumor necrosis factor. Seol et al. (32) suggest that this cell line may activate many resistance mechanisms, one of which may be related to the up-regulation of the defective receptor, TNFRSF10C. However, this was not directly observed in this study.

A synergistic effect between cisplatin and rhTRAIL was observed in our cytotoxicity assays and confirmed by an increase in the *BAX*:*BCL-2* ratio in the TOV-21G (56.42 ratio) and the SKOV-3 (20.34 ratio) cells. In order to determine whether this synergism was due to the gene expression changes, a data clustering analysis was performed. However, genetic signatures were not revealed. Several studies have reported that increased transcription of the *TNFRSF10A* and *TNFRSF10B* receptors is related to the response to the DNA-damaging chemotherapeutic agents such as cisplatin, doxorubicin, and etoposide in several cell types (33,34). Using MTT cytotoxicity assays in the SKOV-3 cells, Cuello et al. (7) demonstrated that combining TRAIL and cisplatin in different doses was more effective than the individual use of the molecules. They also evaluated the mRNA and protein expressions of *TNFRSF10A* and *TNFRSF10B* in response to the combined treatment with TRAIL and cisplatin. Despite up-regulation of these receptor genes after combined treatment, protein expression was not significantly altered. Duiker et al. (19) also evaluated the synergistic effects of cisplatin and rhTRAIL on gene expression in the cisplatin-sensitive (A2780) and the cisplatin-resistant (CP70) OC cell lines. They demonstrated that the synergism between these drugs was associated with changes to *CASPASE*-*8* expression rather than increased *TNFRSF10B* receptor expression. We observed up-regulation of *CASPASE-8* and down-regulation of *TNFRSF10B* in both cell lines treated with a combination of both drugs, corroborating with the data described by Duiker et al. (19).

## CONCLUSION

*CASPASE-8* and *TNFRSF10B* expression may predict the response of OC cell lines to rhTRAIL alone or in combination with cisplatin. Our study suggests that *BCL-2* and *BAX* expression may be helpful in the assessment of the proliferative rate potential of OC cell lines, and highlighted that the development of new therapeutic strategies for the OC should focus on the individual molecular and genetic characteristics of different tumor subtypes. In light of these findings, our future research will aim to explore the exact mechanism behind the synergy between cisplatin and rhTRAIL.

## AUTHOR CONTRIBUTIONS

Braga LC is the main researcher, responsible for the conceptualization, data collection and analysis, funding acquisition, project administration, literature review and manuscript revision.

Gonçales NG was responsible for data analysis, resources, data collection and interpretation and for writing the manuscript original draft. Furtado RS was responsible for the literature review, manuscript revision and submission. Andrade WP was responsible for the data collection and interpretation. Silva LM was responsible for the conceptualization, data analysis, supervision and manuscript revision. Silva Filho AL is the research advisor responsible for the conceptualization, supervision and validation.

## Figures and Tables

**Table 1:** Information about primers used in qRT-PCR.

Gene symbol*	Genetic information	Primers sequence (5′-3′)	Primer concentration (nM)	Melting temperature
TNFRSF10B	NM_003842.5	FW GGGAGCCGCTCATGAGGAAGTTG RV GGCAAGTCTCTCTCCCAGCGTCTC	250FW/200RV	60°C
TNFRSF10C	NM_003841.4	FW GTTTGTTTGAAAGACTTCACTGTG RV GCAGGCGTTTCTGTCTGTGGGAAC	200FW/200RV	60°C
CASPASE-8	NM_001080125.1	FW AGAGCCAGGGTGGTTATTGAA RV GCAGTCTCCGAGTCCCCTA	250FW/200RV	64°C
BCL-2	NM_000633.2	FW GAGTAAATCCATGCACCTAAACC RV TGCAAATTCTACCTTGGAGGG	250FW/200RV	60°C
TBP	NM_003194.5	FW TGCACAGGAGCCAAGAGTGAA RV CACATCACAGCTCCCCACCA	200FW/200RV	60°C
MDM2	NM_001367990.1	FW AGATCCTGAGATTTCCTTAGCTGACT RV TCTCACGAAGGGTCCAGCATCT	250FW/250RV	58°C
TP53TG5	NM_014477.3	FW ACTTGTCGCTCTTGAAGCTA RV GATGCGGAGAATCTTTGGAAC	350FW/300RV	55°C
BAX	NM_138761.4	FW TGCTAGCAAACTGGTGCTCAA RV GCCCATGATGGTTCTGATCAGCT	150FW/100RV	59°C
RPS-26	NG_023201.1	FW CGTGCTTCCCAAGCTGTACGTGA RV CGATTCCGGACTACCTTGCTGTG	200FW/250RV	64°C

* Gene symbol and name according to the HUGO Gene Nomenclature Committee (HGNC) – the European Bioinformatics Institute (EMBL-EBI)

**Table 2:** Concentrations of cisplatin and rhTRAIL tested for the SKOV-3 cell line and cell viabilities obtained.

Proportion	Cisplatin concentration (ug/mL)	rhTRAIL concentration (ng/mL)	Cell viability (%)
1/1	271	196.5	28.49
2/2	135.5	98.2	44.58
3/3	90.3	65.5	64.43
4/4	67	49.1	81.51
5/5	54.2	39.3	56.64
10/10	27.1	19.6	71.38
